# Vaccination Inequities Among Foreign-Born Adults in the United States: The Role of Citizenship, Socioeconomic Disadvantage, and Healthcare Access in the 2024 National Health Interview Survey

**DOI:** 10.3390/tropicalmed11100282

**Published:** 2026-10-05

**Authors:** Gulzar Shah, Indira Karibayeva

**Affiliations:** 1 Department of Health Policy and Community Health, Jiann-Ping Hsu College of Public Health, Georgia Southern University, Statesboro, GA 30460, USA; gshah@georgiasouthern.edu; 2 Department of Research Management, JSC Research Institute of Cardiology and Internal Diseases, Almaty 050012, Kazakhstan

**Keywords:** immigrants, citizenship, vaccination, influenza, pneumococcal, herpes zoster, COVID-19, health insurance, healthcare access, National Health Interview Survey

## Abstract

Foreign-born adults make up a growing and diverse share of the U.S. population. Yet much of what is known about adult vaccination comes from studies conducted before the COVID-19 pandemic. Many focused on a single vaccine or treated immigrants as a single group. We examined influenza, COVID-19, shingles, and pneumococcal vaccination among U.S.-born adults, foreign-born U.S. citizens, and foreign-born noncitizens. We also assessed the extent to which observed differences were attenuated after adjustment for socioeconomic conditions and access to healthcare. Data came from 31,362 adults who participated in the 2024 National Health Interview Survey. We used survey-weighted modified Poisson regression and adjusted sequentially for demographic, socioeconomic, and healthcare access factors. Family income was pooled across ten NCHS imputations. Noncitizens had the lowest unadjusted vaccination prevalence. Influenza vaccination was 32.2% among noncitizens compared with 43.5% among U.S.-born adults. Shingles vaccination rates were 24.3% versus 45.1%, and pneumococcal vaccination rates were 34.5% versus 67.2%. After full adjustment, the influenza difference was no longer evident (adjusted prevalence ratio or aPR 1.07; 95% CI 0.98–1.17). The gap in shingles vaccination narrowed substantially (aPR 0.85; 95% CI 0.71–1.02). In contrast, disparities in pneumococcal vaccination persisted among both noncitizens (aPR 0.70; 95% CI 0.55–0.90) and foreign-born U.S. citizens (aPR 0.85; 95% CI 0.78–0.94). A different pattern emerged for ever receipt of at least one COVID-19 vaccine dose. Both foreign-born groups were more likely than U.S.-born adults to have ever received at least one dose; because this outcome is cumulative receipt since 2020, it does not indicate whether respondents were up to date with current COVID-19 vaccination recommendations. Overall, disparities among foreign-born adults depended on the vaccine being examined. Differences in influenza and shingles vaccination were substantially attenuated after adjustment for socioeconomic and healthcare access characteristics, whereas the pneumococcal difference was not.

## 1. Introduction

Vaccines have dramatically reduced the burden of infectious diseases and continue to play a central role in protecting population health [1,2]. Their importance extends across the lifespan but becomes especially pronounced in adulthood, when age-related changes in immunity, evolving exposure risks, and the presence of chronic health conditions can increase vulnerability to vaccine-preventable diseases and their complications [1,3]. In the United States, the Advisory Committee on Immunization Practices (ACIP) recommends adult vaccines on the basis of age, health conditions, prior vaccination history, and other risk factors, including annual influenza vaccination, COVID-19 vaccination, recombinant zoster vaccination for adults aged 50 years and older, and pneumococcal vaccination for older adults and others with qualifying medical conditions [4,5]. Uptake nevertheless lags behind national goals for several adult immunizations, and substantial differences in coverage continue to be observed across population groups [6,7,8,9].

Foreign-born adults represent a growing segment of the U.S. population. More than 47 million foreign-born individuals live in the United States, representing roughly 14% of the population [10]. Foreign-born adults in the United States represent a diverse population with a wide range of immigration experiences and legal statuses, including naturalized citizens, lawful permanent residents, temporary residents, refugees, and individuals without authorized immigration status [10,11]. Differences in socioeconomic conditions, English-language use, health insurance coverage, and access to healthcare services can be substantial across these groups [11,12,13]. Such differences are particularly relevant to adult vaccination because most recommended vaccines are delivered through routine interactions with the healthcare system.

Previous studies have documented lower uptake of several routinely recommended vaccines among foreign-born adults. Using data from the 2012 National Health Interview Survey (NHIS), Lu et al. reported lower vaccination coverage among foreign-born adults for influenza, pneumococcal, shingles, tetanus-containing, hepatitis B, and human papillomavirus vaccines [14]. Coverage also varied according to citizenship status, length of residence in the United States, and language of interview [14]. Other studies similarly reported lower influenza vaccination among some foreign-born populations [15]. Disparities have not been limited to influenza vaccination. Lower uptake of pneumococcal and shingles vaccines has also been observed among immigrant and racial or ethnic minority populations [14,16].

Several factors may contribute to these disparities. Compared with U.S.-born adults, foreign-born adults, particularly noncitizens, are more likely to be uninsured and to experience challenges accessing healthcare services [12,13,17]. Individuals without a usual source of care may have fewer opportunities to receive preventive services, discuss vaccination needs with healthcare providers, or obtain vaccine recommendations during routine visits. Cost concerns, language barriers, unfamiliarity with the U.S. healthcare system, and difficulties navigating available services may further limit access to vaccination [17,18,19]. Citizenship status may also shape access to preventive care, because eligibility for health insurance programs and for other publicly funded services differs across citizenship groups: naturalized citizens generally have greater access to health insurance and healthcare services than noncitizens, although considerable variation remains within both groups [11,12,13]. These structural factors can shape opportunities for vaccination independently of individual attitudes or preferences, so treating all foreign-born adults as a single population may obscure meaningful differences in vaccination opportunity and healthcare access.

The COVID-19 pandemic introduced a distinctive context for adult vaccination. Immigrant communities experienced substantial exposure to COVID-19 and, in many settings, faced barriers to testing, treatment, and healthcare services [18,19,20]. At the same time, COVID-19 vaccines were delivered through channels that differed from those used for many routine adult vaccines, including pharmacies, workplaces, public health departments, mass vaccination sites, mobile clinics, and community-based organizations, often supported by trusted messengers and culturally and linguistically appropriate outreach [20,21]. Patterns of COVID-19 vaccination among foreign-born adults may therefore differ from those observed for more traditionally delivered vaccines such as influenza, shingles, and pneumococcal vaccines.

Despite growing research on vaccination among immigrant populations, important gaps remain. Much of the available national evidence predates the COVID-19 pandemic, focuses on individual vaccines, or treats foreign-born adults as a homogeneous population despite differences in citizenship status and healthcare access [14,15,16]. Less is known about the extent to which socioeconomic disadvantage and healthcare access statistically account for vaccination differences between foreign-born U.S. citizens and noncitizens using contemporary nationally representative data. It is also unclear whether these associations vary according to health insurance coverage and family income. Duration of residence in the United States may also influence vaccination uptake, but prior findings have been inconsistent across vaccines and populations [14,15].

The purpose of this study was to examine vaccination uptake among U.S.-born adults, foreign-born U.S. citizens, and foreign-born noncitizens. We assessed four routinely recommended adult vaccines: influenza, COVID-19, shingles, and pneumococcal vaccination. We examined the extent to which differences in vaccination uptake were attenuated after adjustment for demographic characteristics, socioeconomic disadvantage, and healthcare access. We also evaluated whether these associations varied according to health insurance coverage and family income, and whether vaccination patterns among foreign-born adults varied according to length of residence in the United States. Examining multiple vaccines within a contemporary national population allows a more comprehensive assessment of adult vaccination inequities and highlights where gaps in coverage remain as well as where patterns diverge across vaccines.

## 2. Materials and Methods

### 2.1. Study Design and Data Source

We conducted a cross-sectional analysis of the 2024 National Health Interview Survey (NHIS), a continuous, nationally representative household survey of the civilian, non-institutionalized population of the United States administered by the National Center for Health Statistics (NCHS) [22,23]. The NHIS uses a multistage, stratified, clustered probability sampling design; within each sampled household, one adult aged ≥18 years (the Sample Adult) is randomly selected to complete a detailed interview. Data are collected by trained interviewers throughout the calendar year using computer-assisted personal and telephone interviewing. We used the 2024 Sample Adult public-use file (32,629 respondents) linked, by household identifier, to the 2024 Sample Adult multiply imputed family income file, which provides 10 imputed values of the family income-to-poverty ratio for every respondent. Analyses used the NCHS-supplied final annual Sample Adult weight (WTFA_A), pseudo-stratum (PSTRAT), and pseudo-primary sampling unit (PPSU) variables. The survey weights account for the probability of selection, non-response, and post-stratification to U.S. Census Bureau population controls for age, sex, race/ethnicity, and geography, so that weighted estimates are representative of the U.S. adult population.

### 2.2. Study Population and Analytic Samples

All Sample Adults with valid information on nativity and citizenship were eligible (*n* = 31,362; 96.1% of the file). Because the four vaccines have different age-based indications, we defined an outcome-specific analytic population for each vaccine rather than a single common denominator. Influenza and COVID-19 vaccination were analyzed among all adults aged ≥18 years, and shingles vaccination among adults aged ≥50 years, consistent with the routine age-based recommendation for recombinant zoster vaccine [24]. Pneumococcal vaccination was analyzed among adults aged ≥65 years. Although the Advisory Committee on Immunization Practices (ACIP) lowered the age threshold for routine pneumococcal vaccination from 65 to 50 years in late October 2024 [25], we deliberately retained a single ≥65-year denominator for the entire survey year: the ≥65-year recommendation was in force for approximately 10 of the 12 months of data collection, and the NHIS “ever vaccinated” item cannot distinguish vaccination received under the new recommendation from earlier vaccination. Applying the ≥50-year threshold only to respondents interviewed after the change would have produced a denominator that depended on interview timing and was not comparable across the annual sample, and would have combined newly eligible adults aged 50–64 years—who had had at most two months to be vaccinated under the new recommendation—with adults aged ≥65 years who had been eligible for decades. The likely direction of any bias introduced by this choice can be anticipated. Because foreign-born noncitizens are considerably younger than U.S.-born adults, a ≥50-year denominator would have added proportionally more foreign-born than U.S.-born adults who had had at most two months of eligibility and whose vaccination prevalence would therefore have been very low. Restricting the analysis to adults aged ≥65 years is thus expected to understate rather than exaggerate the foreign-born pneumococcal deficit, and the estimates reported here should be regarded as conservative in that respect. Adults aged 50–64 years are retained in the shingles analysis, and the influence of the pneumococcal denominator can be examined in future survey years.

We excluded respondents with missing or invalid outcome data (refused, not ascertained, don’t know) from the corresponding outcome analysis. Regression models used complete-case analytic samples that additionally required non-missing covariate data; because family income is fully imputed by NCHS, the analytic sample for each model was identical across the 10 imputations. Sample sizes for each model are reported alongside the estimates and in Supplementary Table S1.

### 2.3. Primary Exposure: Nativity and Citizenship

The primary exposure combined nativity (NATUSBORN_A: born in the United States or a U.S. territory versus born elsewhere) and citizenship (CITZNSTP_A: U.S. citizen versus not a U.S. citizen) into three mutually exclusive groups: (1) U.S.-born adults (reference), (2) foreign-born U.S. citizens (naturalized citizens), and (3) foreign-born noncitizens. The NHIS does not collect immigration or visa status; the noncitizen category therefore comprises lawful permanent residents, temporary visa holders, and undocumented residents. Among foreign-born adults, duration of U.S. residence (YRSINUS_A) was collapsed from the five NHIS categories into <5 years, 5–14 years, and ≥15 years (reference) to maintain adequate cell sizes.

### 2.4. Outcomes

Four self-reported vaccination outcomes were analyzed, each coded as a binary indicator (yes = 1, no = 0): (1) receipt of an influenza vaccine during the past 12 months (SHTFLU12M_A), the primary outcome; (2) ever receipt of at least one dose of COVID-19 vaccine (SHTCVD191_A); (3) ever having received a shingles (herpes zoster) vaccine (SHTSHINGL1_A); and (4) ever having received a pneumococcal vaccine (SHTPNUEV_A). All variable names are reproduced exactly as they appear in the 2024 NHIS Sample Adult public-use file and were re-verified against the 2024 public-use codebook before analysis; the pneumococcal item is spelled SHTPNUEV_A in the NCHS documentation, and that spelling is retained here [22]. Influenza vaccination captures current-season uptake and therefore reflects contemporary access to and engagement with preventive services. In contrast, the COVID-19 measure captures cumulative historical uptake since 2020 and does not indicate whether the respondent is up to date with current recommendations; it was retained as a contrasting outcome that reflects the distinctive delivery context of the pandemic-era vaccination program. To avoid any implication that it reflects current coverage, this outcome is described throughout as ever receipt of at least one COVID-19 vaccine dose. Shingles and pneumococcal vaccination are likewise lifetime (“ever”) measures. Variable definitions are summarized in Supplementary Table S2.

### 2.5. Covariates

Covariates were selected a priori on the basis of prior literature on immigrant health and vaccination and were organized into three conceptual blocks to support sequential adjustment. Demographic and geographic characteristics comprised age group (18–29, 30–49, 50–64, 65–74, ≥75 years), sex (female, male), race/ethnicity (NHIS single-variable public-use recode HISPALLP_A: non-Hispanic White [reference], Hispanic, non-Hispanic Black, non-Hispanic Asian, and non-Hispanic other or multiracial), U.S. Census region (Northeast, Midwest, South, West), and the 2023 NCHS urban–rural classification (large central metropolitan, large fringe metropolitan, medium/small metropolitan, non-metropolitan) [26]. Socioeconomic and marginalization characteristics comprised educational attainment (less than high school, high school diploma or GED, some college or associate degree, bachelor’s degree or higher), family income as a ratio of the federal poverty level (FPL; ≥400% [reference], 200–399%, 100–199%, and <100% FPL), and whether a language other than English is spoken at home (LANGHM_A). Family income-to-poverty ratio was derived from the NCHS multiply imputed variable RATCAT_A in each of the 10 imputed data sets, as described below. Healthcare access characteristics comprised health insurance coverage at the time of interview (insured versus uninsured, using the NCHS-edited coverage indicator NOTCOV_A), having a usual place for care other than an emergency department (USUALPL_A), any cost-related care barrier in the past 12 months (delaying medical care [MEDDL12M_A] or needing but not obtaining medical care [MEDNG12M_A] because of cost), and having seen a doctor or other health professional within the past 12 months (LASTDR_A). For effect-modification analyses, family income was dichotomized as <200% versus ≥200% FPL within each imputed data set.

### 2.6. Statistical Analysis

All analyses accounted for the complex survey design using the final annual Sample Adult weight and the pseudo-stratum and pseudo-PSU variables, with Taylor-series linearization for variance estimation and the “adjust” option for strata containing a single PSU. Following NCHS guidance for subpopulation analysis [27], the survey design object was constructed on the full Sample Adult file and each outcome-specific or subgroup analysis was performed by subsetting that design, so that full design information was retained for domain variance estimation. Sample characteristics are presented as unweighted counts with survey-weighted percentages, and differences across nativity/citizenship groups were assessed with design-adjusted Rao–Scott chi-square tests (F-statistic) [28]. Survey-weighted vaccination prevalence and 95% confidence intervals (CIs) were estimated for each nativity/citizenship group within each outcome-specific population using logit-transformed CIs.

Because all four vaccination outcomes are common (prevalence >10%), odds ratios from logistic regression would overstate relative differences [29,30]. We therefore estimated adjusted prevalence ratios (aPRs) with 95% CIs using survey-weighted modified Poisson regression (generalized linear model with a log link and quasi-Poisson variance, with design-based robust standard errors) [29,30]. Relative and absolute measures are reported in parallel throughout, because a prevalence ratio can make a small absolute gap appear large when an outcome is uncommon, whereas a prevalence difference can make a large relative gap appear negligible when an outcome is common; presenting both prevents either impression from standing alone. To examine how much of the observed inequity was attenuated after sequential adjustment for successive blocks of covariates, three nested models were fitted for each outcome: Model 1 adjusted for the demographic and geographic block (age group, sex, race/ethnicity, region, and urbanicity); Model 2 additionally adjusted for the socioeconomic and marginalization block (education, family income-to-poverty ratio, and language spoken at home); and Model 3, the fully adjusted model, additionally adjusted for the healthcare access block (insurance, usual place for care, cost-related care barrier, and healthcare contact in the past year). Given the cross-sectional design, changes across models are described as attenuation under sequential adjustment rather than as formal mediation. Where reported, the proportional attenuation of the Model 1 association after additional adjustment was calculated on the log-prevalence-ratio scale as [ln(aPR_1_) − ln(aPR_k_)]/ln(aPR_1_). These proportions are descriptive comparisons of coefficients across nested models and are not estimates of a mediated proportion; they should not be interpreted as such.

Because prevalence ratios convey relative but not absolute inequities, we also used model-based marginal standardization (g-computation) [31] from the fully adjusted model to estimate the adjusted (standardized) vaccination prevalence for each nativity/citizenship group and the adjusted prevalence difference (aPD, in percentage points) relative to U.S.-born adults. For each group, the fully adjusted model was used to predict the probability of vaccination for every respondent in the analytic sample with nativity/citizenship set to that group and all other covariates held at their observed values; predictions were then averaged over the survey-weighted covariate distribution. Standard errors for standardized prevalences and differences were derived by the delta method from the design-based variance–covariance matrix of the fitted model.

### 2.7. Multiply Imputed Family Income

Family income in the NHIS is subject to substantial item non-response and is multiply imputed by NCHS, which releases 10 completed income data sets and recommends that analyses involving income be repeated in each imputed data set and combined, so that between-imputation variability is reflected in the standard errors [32]. We followed this recommendation for every analysis that included family income. The 10 imputed income files were merged to the Sample Adult file by household identifier (HHX); merge integrity was verified for each imputation (Supplementary Table S3). Model 1, which does not include income, was fitted once. Models 2 and 3, the marginally standardized prevalences and aPDs, the duration-of-residence models, and both prespecified interaction analyses (including the family income interaction, whose modifier itself varies across imputations) were fitted separately in each of the 10 imputed data sets and combined with Rubin’s rules [33]: point estimates were averaged across imputations, and the total variance was computed as the within-imputation variance plus (1 + 1/10) times the between-imputation variance. Confidence intervals and *p* values used a t reference distribution with degrees of freedom from the Barnard–Rubin small-sample adjustment [34], taking the design-based residual degrees of freedom of the complete-data models as the complete-data degrees of freedom. Marginally standardized prevalences and differences were computed within each imputation with delta-method variances and then pooled in the same way. Table 1 percentages for the income-to-poverty categories are likewise pooled across imputations, with the unweighted counts shown from the first imputation; a single-imputation Rao–Scott test is not reported for that block because it would not reflect imputation uncertainty. Descriptive analyses not involving income used the Sample Adult file directly, because all non-income variables are identical across imputations.

### 2.8. Effect Modification and Supplementary Analyses

Two effect modifiers were prespecified—health insurance status and low family income (<200% versus ≥200% FPL)—and were examined for the two routinely recommended age-based outcomes with the largest analytic samples: influenza vaccination (adults ≥ 18 years) and shingles vaccination (adults ≥ 50 years). Modification was assessed by adding a product term between nativity/citizenship and the modifier to the fully adjusted model (with the corresponding main-effect covariate retained and, for income, the four-level poverty variable replaced by the binary indicator). Because the interaction models were fitted in all 10 imputed data sets, modification was tested with a joint Wald test of the Rubin-pooled interaction coefficients using the pooled variance–covariance matrix, referred to an F distribution with the number of interaction terms as numerator degrees of freedom and the smallest term-specific Barnard–Rubin degrees of freedom as denominator degrees of freedom. Marginally standardized vaccination prevalence with pooled 95% CIs was estimated for each combination of nativity/citizenship group and modifier level. Among foreign-born adults only, the association between duration of U.S. residence and influenza and COVID-19 vaccination was estimated with fully adjusted modified Poisson models; duration analyses were restricted to these two outcomes because the numbers of recently arrived immigrants aged ≥50 and ≥65 years were insufficient for stable age-specific shingles and pneumococcal models. Restricting effect-modification testing to two prespecified modifiers and two outcomes limited the number of statistical tests.

### 2.9. Missing Data

Apart from family income, which is fully imputed by NCHS, missingness for all analysis variables was low (≤3.9% of Sample Adults for each variable; Supplementary Table S4). The 41.7% without a recorded shingles outcome in Supplementary Table S4 largely reflects structural missingness from the NHIS skip pattern, because the shingles question is administered only to adults aged ≥50 years and younger immunocompromised adults, rather than item non-response; similarly, years lived in the United States is asked only of foreign-born respondents. Complete-case analysis was used for the non-income covariates; the fully adjusted models retained ≥99.2% of eligible respondents with a valid outcome for every vaccine (Supplementary Table S1).

### 2.10. Software and Reporting

Statistical analyses were conducted using R (version 4.5.1; R Foundation for Statistical Computing, Vienna, Austria) [35], with the survey package for design-based analyses [36,37,38] and the mitools package for combining estimates across imputations [39]. Two-sided *p* values < 0.05 were considered statistically significant, and 95% CIs are reported throughout. The study is reported in accordance with the STROBE guidelines for cross-sectional studies [40]. The NHIS is approved by the NCHS Research Ethics Review Board, and all respondents provide informed consent; this secondary analysis of de-identified public-use data did not require additional ethical review.

## 3. Results

### 3.1. Characteristics of the Study Population

Of 32,629 Sample Adults, 31,362 (96.1%) had valid nativity and citizenship information: 26,359 U.S.-born adults, 2920 foreign-born U.S. citizens, and 2083 foreign-born noncitizens (Table 1). The three groups differed on every characteristic examined (all *p* ≤ 0.003). Foreign-born noncitizens were markedly younger than the other groups (50.4% aged 30–49 years and 7.1% aged ≥65 years, versus 31.8% and 24.2% of U.S.-born adults), whereas foreign-born U.S. citizens were older (27.4% aged ≥65 years). Most noncitizens were Hispanic (62.2%), and 19.5% were non-Hispanic Asian; among foreign-born U.S. citizens, 33.9% were Hispanic and 29.2% non-Hispanic Asian, compared with 10.9% and 1.7% of U.S.-born adults. Noncitizens experienced substantially greater socioeconomic disadvantage: 32.8% had less than a high-school education (versus 7.4% of U.S.-born adults and 13.2% of foreign-born U.S. citizens), 52.5% lived in families with incomes below 200% of the FPL (versus 24.6% and 26.6%; percentages pooled across the 10 income imputations), and 88.4% spoke a language other than English at home (versus 9.8% and 72.8%). By contrast, foreign-born U.S. citizens were the most highly educated group (39.9% with a bachelor’s degree or higher) and had an income distribution similar to that of U.S.-born adults (42.8% versus 45.2% at ≥400% FPL).

Healthcare access was markedly poorer among noncitizens. One-third (33.2%) were uninsured, compared with 6.8% of U.S.-born adults and 6.4% of foreign-born U.S. citizens; 23.4% had no usual place for care (versus 8.4% and 7.5%); 13.5% reported a cost-related barrier to care in the past 12 months (versus 9.9% and 8.0%); and 28.4% had not seen a doctor or other health professional in the past year (versus 13.6% and 12.4%). Foreign-born adults of both citizenship groups were concentrated in large central metropolitan areas (48.3% of U.S. citizens and 52.0% of noncitizens, versus 27.1% of U.S.-born adults) and in the West and were rarely resident in non-metropolitan areas (3.4% and 5.3%, versus 16.7%).

### 3.2. Survey-Weighted Vaccination Prevalence

Unadjusted vaccination prevalence differed significantly across nativity/citizenship groups for all four vaccines (all *p* < 0.001; Table 2). Foreign-born noncitizens had the lowest prevalence of influenza vaccination in the past 12 months (32.2%; 95% CI, 29.8–34.7), compared with 43.5% (42.6–44.4) among U.S.-born adults and 49.5% (47.2–51.8) among foreign-born U.S. citizens, who had the highest prevalence. Among adults aged ≥50 years, 24.3% (20.3–28.8) of noncitizens had ever received a shingles vaccine, compared with 45.1% (43.9–46.2) of U.S.-born adults and 43.2% (40.1–46.4) of foreign-born U.S. citizens. The largest absolute gap was observed for pneumococcal vaccination among adults aged ≥65 years: 34.5% (26.7–43.2) of noncitizens had ever been vaccinated, approximately half the prevalence among U.S.-born adults (67.2%; 65.9–68.4), with foreign-born U.S. citizens intermediate (55.7%; 51.8–59.5); this estimate for noncitizens rests on only 160 unweighted respondents and is correspondingly imprecise. The pattern for COVID-19 vaccination was reversed: ever receipt of at least one dose was higher among foreign-born U.S. citizens (88.4%; 86.9–89.9) and noncitizens (82.6%; 80.6–84.5) than among U.S.-born adults (75.6%; 74.7–76.5).

### 3.3. Attenuation of Inequities Under Sequential Adjustment

Sequentially adjusted prevalence ratios, with Models 2 and 3 pooled across the 10 income imputations, are presented in Supplementary Table S5. After adjustment for demographic and geographic characteristics only (Model 1), foreign-born noncitizens remained significantly less likely than U.S.-born adults to have received influenza (aPR 0.84; 95% CI, 0.77–0.92), shingles (aPR 0.64; 0.54–0.77), and pneumococcal (aPR 0.57; 0.45–0.73) vaccination, and foreign-born U.S. citizens were less likely to have received pneumococcal vaccination (aPR 0.83; 0.76–0.91). Adding the socioeconomic block (Model 2) attenuated the noncitizen deficit for each of these vaccines—by approximately 52% for influenza (aPR 0.92; 0.84–1.00; *p* = 0.060), 39% for shingles (aPR 0.76; 0.63–0.91), and 18% for pneumococcal vaccination (aPR 0.63; 0.49–0.81) on the log-prevalence-ratio scale—but the shingles and pneumococcal deficits remained statistically significant. Adding the healthcare access block (Model 3) produced further attenuation, such that there was no longer evidence of lower influenza vaccination among foreign-born noncitizens compared with U.S.-born adults (aPR 1.07; 0.98–1.17); the shingles deficit was reduced to a non-significant 15% relative deficit (aPR 0.85; 0.71–1.02), and the pneumococcal deficit was reduced to 30% (aPR 0.70; 0.55–0.90) but persisted. Cumulatively, demographic-adjusted noncitizen deficits were attenuated by approximately 64% for shingles and 37% for pneumococcal vaccination after full adjustment. The pneumococcal deficit among foreign-born U.S. citizens was essentially unchanged across models (aPR 0.83, 0.86, and 0.85 in Models 1–3), indicating that it was not statistically accounted for by the measured socioeconomic or access variables. For COVID-19 vaccination, the advantage of foreign-born adults increased with adjustment, most notably among noncitizens (aPR 1.08 in Model 1, 1.13 in Model 2, and 1.19 in Model 3). All attenuation percentages reported here are descriptive comparisons of prevalence ratios across nested models fitted in a cross-sectional sample; they quantify how much an association changes when further covariates are added and must not be read as proportions mediated.

### 3.4. Fully Adjusted Associations

**Figure 1:**
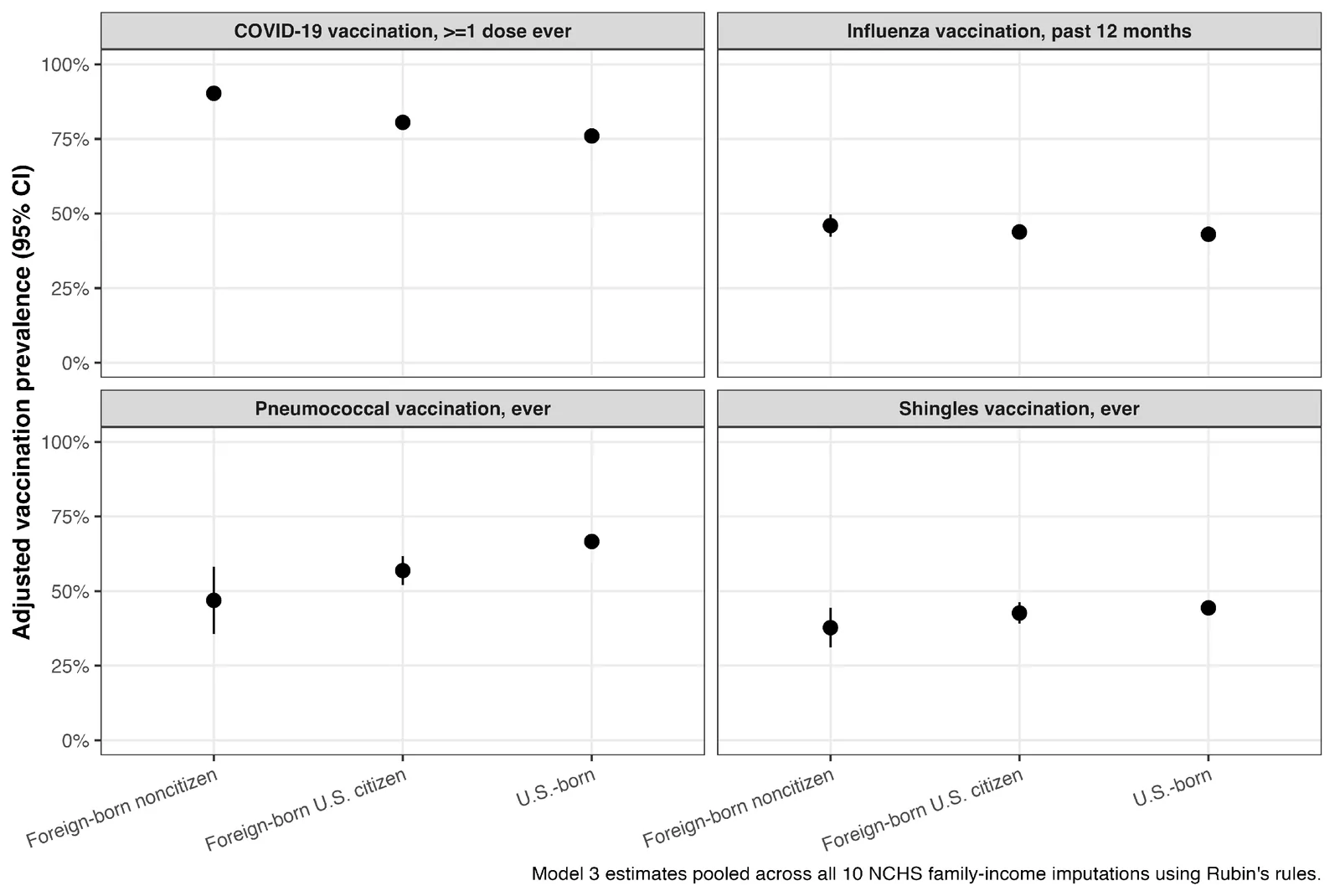
Adjusted prevalence of vaccination by nativity and citizenship status, U.S. adults, 2024. Points are marginally standardized vaccination prevalences with 95% confidence intervals from fully adjusted survey-weighted modified Poisson models (adjusted for age group, sex, race/ethnicity, Census region, urbanicity, education, family income-to-poverty ratio, language spoken at home, health insurance, usual place for care, cost-related care barrier in the past 12 months, and healthcare contact in the past year), pooled across the 10 NCHS family income imputations using Rubin’s rules. Influenza and COVID-19 panels include adults aged ≥18 years; the shingles panel includes adults aged ≥50 years; the pneumococcal panel includes adults aged ≥65 years.

**Figure 2:**
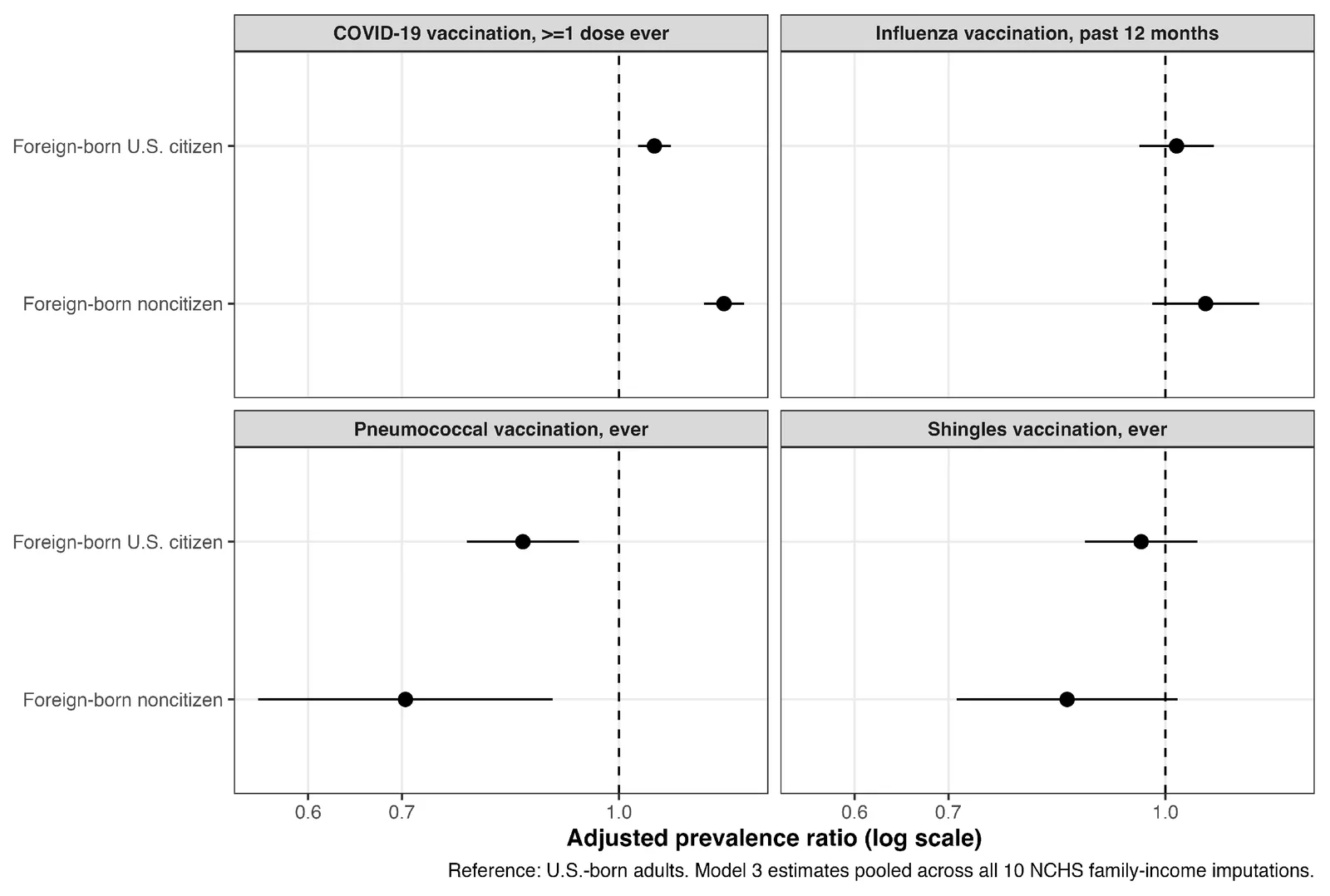
Fully adjusted prevalence ratios for vaccination uptake among foreign-born U.S. citizens and foreign-born noncitizens relative to U.S.-born adults (reference, dashed line), U.S. adults, 2024. Points are multiply-imputation-pooled adjusted prevalence ratios with 95% confidence intervals plotted on a logarithmic scale; adjustment set as in Figure 1.

Fully adjusted associations, together with marginally standardized adjusted prevalences and absolute prevalence differences pooled across imputations, are presented in Table 3 and Figure 1 and Figure 2; unweighted numbers of respondents in each nativity/citizenship group are shown in Table 3 alongside the estimates. After full adjustment for demographic, socioeconomic, and healthcare access characteristics, influenza vaccination did not differ between U.S.-born adults (adjusted prevalence 43.0%; 95% CI, 42.2–43.9), foreign-born U.S. citizens (43.8%; 41.4–46.3; aPR 1.02, 0.96–1.08; aPD 0.8 percentage points, −1.9 to 3.5), and foreign-born noncitizens (46.0%; 42.2–49.7; aPR 1.07, 0.98–1.17; aPD 2.9 percentage points, −1.1 to 7.0).

For shingles vaccination among adults aged ≥50 years, the adjusted prevalence was 44.4% (43.3–45.5) among U.S.-born adults, 42.7% (39.1–46.2) among foreign-born U.S. citizens (aPR 0.96; 0.88–1.05), and 37.8% (31.2–44.4) among noncitizens (aPR 0.85; 0.71–1.02; aPD −6.6 percentage points, −13.5 to 0.3; *p* = 0.081). For pneumococcal vaccination among adults aged ≥65 years, significant deficits persisted for both foreign-born groups: the adjusted prevalence was 66.6% (65.3–67.9) among U.S.-born adults, 56.9% (52.1–61.7) among foreign-born U.S. citizens (aPR 0.85; 0.78–0.94; aPD −9.7 percentage points, −15.1 to −4.4; *p* < 0.001), and 46.9% (35.7–58.1) among noncitizens (aPR 0.70; 0.55–0.90; aPD −19.7 percentage points, −31.1 to −8.3; *p* = 0.005). Adjusted pneumococcal vaccination prevalence was therefore progressively lower among U.S.-born adults, foreign-born U.S. citizens, and foreign-born noncitizens. The noncitizen estimate is based on only 160 unweighted respondents aged ≥65 years, and its wide confidence interval should be kept in view when this comparison is interpreted.

In contrast, both foreign-born groups were significantly more likely than U.S.-born adults to have ever received at least one dose of COVID-19 vaccine after full adjustment: adjusted prevalence was 76.0% (75.2–76.8) among U.S.-born adults, 80.5% (78.6–82.4) among foreign-born U.S. citizens (aPR 1.06; 1.03–1.09; aPD 4.6 percentage points, 2.4 to 6.7), and 90.3% (87.7–92.9) among noncitizens (aPR 1.19; 1.15–1.23; aPD 14.3 percentage points, 11.4 to 17.2; both *p* < 0.001).

### 3.5. Effect Modification by Health Insurance and Family Income

**Figure 3:**
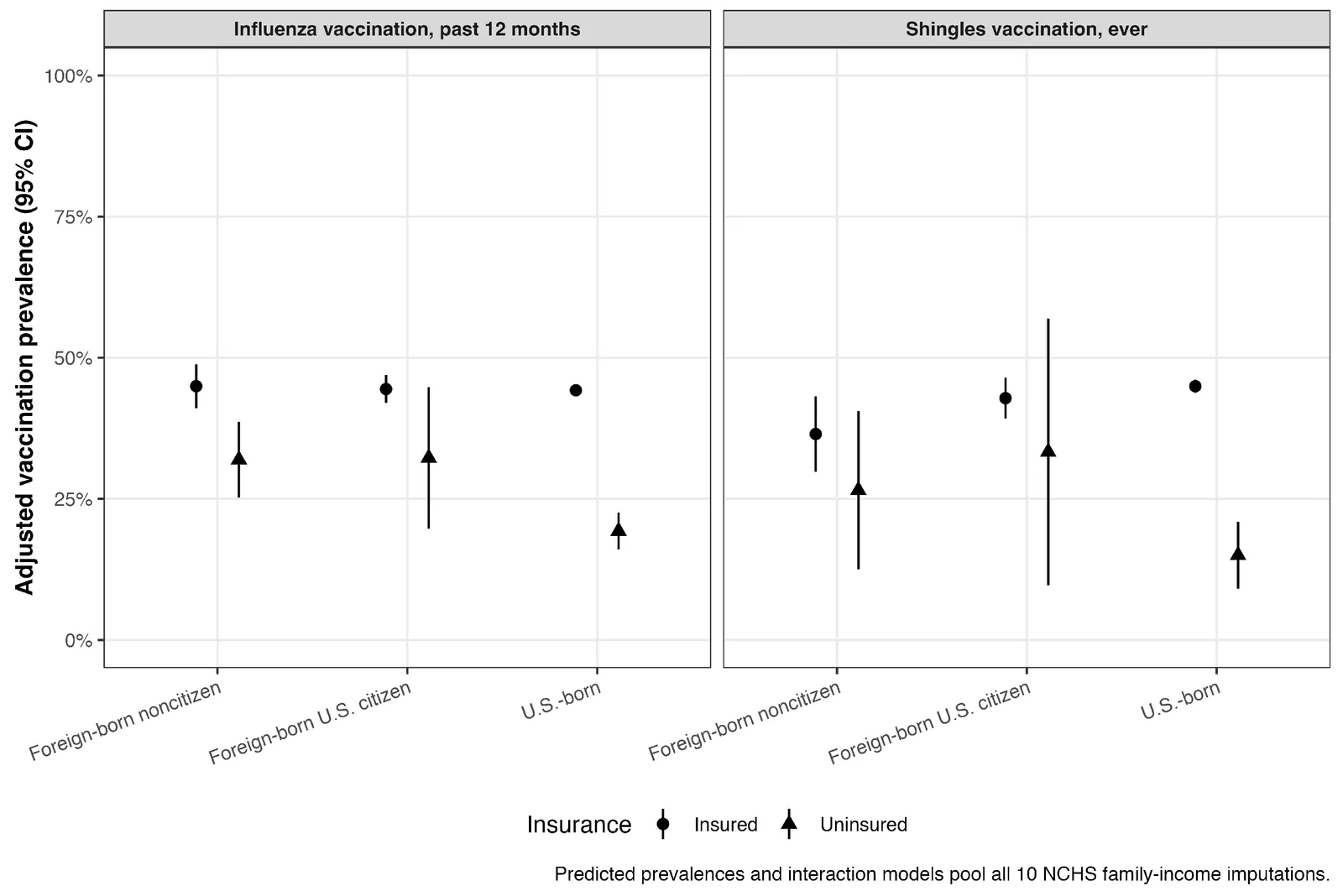
Effect modification by health insurance status of nativity/citizenship inequities in influenza vaccination (adults ≥ 18 years) and shingles vaccination (adults ≥ 50 years), U.S. adults, 2024. Points are marginally standardized vaccination prevalences with 95% confidence intervals for insured (circles) and uninsured (triangles) adults in each nativity/citizenship group, from fully adjusted models that included a nativity/citizenship × insurance product term, fitted in each of the 10 income imputations and pooled using Rubin’s rules. Global interaction *p* values from joint Wald tests of the pooled interaction coefficients: influenza *p* < 0.001; shingles *p* = 0.022 (Supplementary Table S6a).

In pooled multiply imputed interaction models, nativity/citizenship inequities in influenza vaccination differed significantly by both health insurance status (global interaction *p* < 0.001) and family income (*p* < 0.001) (Supplementary Table S6a; Figure 3). Among insured adults, adjusted influenza vaccination prevalence was similar across groups (44.2% [43.3–45.1] U.S.-born, 44.5% [42.0–46.9] foreign-born U.S. citizens, and 45.0% [41.1–48.9] noncitizens). Among uninsured adults, however, prevalence was substantially lower overall and lowest among U.S.-born adults (19.3%; 16.0–22.6), with uninsured foreign-born U.S. citizens (32.3%; 19.7–44.8) and noncitizens (32.0%; 25.3–38.7) each vaccinated at roughly 1.7 times the U.S.-born rate (Supplementary Table S6b). Thus, the absolute insurance-related gap in influenza vaccination was approximately 25 percentage points among U.S.-born adults but approximately 12–13 percentage points among foreign-born adults. A similar pattern was observed for income: among adults with family incomes ≥200% FPL, adjusted prevalence was 44.3% (43.3–45.3) among U.S.-born adults, 42.9% (40.3–45.6) among foreign-born U.S. citizens, and 41.6% (37.3–45.9) among noncitizens, whereas among adults with incomes <200% FPL the ordering was reversed—38.7% (37.1–40.4), 45.5% (41.1–50.0), and 49.6% (43.6–55.6), respectively—such that low income was associated with lower influenza vaccination among U.S.-born adults but not among foreign-born adults. This reversal rests on small subgroups: 2072 foreign-born noncitizens contributed to the influenza analysis in total, of whom 52.5% had family incomes below 200% FPL, and the corresponding confidence interval spans 12 percentage points (49.6%; 43.6–55.6). The reversal should therefore be regarded as a signal warranting replication rather than as a precisely estimated difference.

For shingles vaccination, the nativity/citizenship association also varied by insurance status (interaction *p* = 0.022) but not by family income (*p* = 0.168). Among insured adults aged ≥50 years, the adjusted prevalence was 45.0% (43.8–46.1) among U.S.-born adults, 42.9% (39.2–46.5) among foreign-born U.S. citizens, and 36.5% (29.8–43.2) among noncitizens. Among uninsured adults, adjusted prevalence was low in all groups and imprecisely estimated because of small numbers (15.0% [9.1–20.9] U.S.-born, 33.3% [9.7–56.9] foreign-born U.S. citizens, and 26.5% [12.5–40.6] noncitizens). These uninsured strata are sparsely populated; the estimate for uninsured foreign-born U.S. citizens spans a confidence interval of more than 47 percentage points and is unstable, so it should be read as evidence of imprecision rather than as an interpretable point difference. Among adults with family incomes <200% FPL, shingles vaccination prevalence was similar across the three groups (36.1%, 37.3%, and 36.4%), whereas among higher-income adults noncitizens had a lower prevalence (35.1%; 26.8–43.4) than U.S.-born adults (46.7%; 45.4–48.0) (Supplementary Table S6b).

### 3.6. Duration of U.S. Residence Among Foreign-Born Adults

Among foreign-born adults included in the complete-case duration analyses (n = 4846 for influenza and 4851 for COVID-19), fully adjusted influenza vaccination prevalence was 42.7% (40.9–44.6) among those resident in the United States for ≥15 years, 39.2% (34.6–43.9) among those resident 5–14 years (aPR 0.92; 0.80–1.05), and 38.3% (31.5–45.2) among those resident <5 years (aPR 0.90; 0.74–1.08); neither difference was statistically significant (Supplementary Table S7; Supplementary Figure S1). For ever receipt of at least one COVID-19 vaccine dose, the most recently arrived immigrants (<5 years) had a higher adjusted prevalence (93.9%; 90.0–97.8) than those resident ≥15 years (84.9%; 83.4–86.5; aPR 1.11; 1.05–1.16; *p* < 0.001), with those resident 5–14 years intermediate (87.4%; 84.6–90.2; aPR 1.03; 0.99–1.07).

## 4. Discussion

This study examined differences in adult vaccination among U.S.-born adults, foreign-born U.S. citizens, and foreign-born noncitizens using nationally representative 2024 NHIS data. Several findings stand out. Foreign-born noncitizens had substantially lower crude uptake of influenza, shingles, and pneumococcal vaccines than U.S.-born adults. These differences were substantially attenuated after adjustment for socioeconomic conditions and healthcare access. The influenza difference was no longer evident after full adjustment, and the shingles difference was also substantially reduced. Pneumococcal vaccination was different. A sizeable gap remained among both foreign-born U.S. citizens and noncitizens. Ever receipt of at least one COVID-19 vaccine dose showed a different pattern altogether: foreign-born adults were more likely than U.S.-born adults to report having ever been vaccinated. Taken together, these findings show that vaccination inequities among foreign-born adults vary by vaccine. They cannot be described simply as lower vaccination among immigrants.

Our influenza findings are generally consistent with earlier national studies showing lower vaccination among foreign-born adults and non-U.S. citizens [14,15]. Lu et al. found lower uptake of several adult vaccines among foreign-born adults in the 2012 NHIS. Coverage was generally lower among noncitizens and recent immigrants [14]. Chuey et al. later reported lower influenza vaccination among foreign-born adults and non-U.S. citizens across several influenza seasons [15]. Our findings add to this literature in an important way. In the 2024 data, much of the initial influenza difference among noncitizens was attenuated after socioeconomic conditions and healthcare access were added to the models. Noncitizens were more likely to be uninsured, lack a usual place for care, and have less healthcare contact. These characteristics statistically accounted for a substantial part of the observed influenza vaccination gap. Because the data are cross-sectional and all covariates were measured at the 2024 interview, this pattern does not establish mediation or causality.

A similar pattern was seen for shingles vaccination. The crude difference among noncitizens was large, but it became much smaller after adjustment. Previous research has also documented lower shingles vaccination among foreign-born adults [14]. A systematic review and meta-analysis of vaccination among refugees, immigrants, and migrants in the United States found lower coverage than in the general population and identified poor access to medical care, gaps in knowledge, and distrust of the medical system as key barriers [41].

Our findings are consistent with this broader evidence. For influenza and shingles, access to care and socioeconomic conditions appear to be an important part of the picture. Pneumococcal vaccination was the clearest exception. Among adults aged 65 years and older, only 34.5% of noncitizens reported ever receiving pneumococcal vaccination, compared with 67.2% of U.S.-born adults. After full adjustment, standardized prevalence remained 46.9% among noncitizens compared with 66.6% among U.S.-born adults, an adjusted difference of nearly 20 percentage points. Foreign-born citizens also remained less likely to be vaccinated. Earlier NHIS research found a similar pneumococcal disadvantage among older foreign-born adults [14]. The persistence of this difference more than a decade later is concerning.

Several explanations for the pneumococcal finding are plausible, although none could be tested with these data and all should be regarded as hypotheses rather than demonstrated mechanisms. Older foreign-born adults may have vaccination histories that span more than one country, and doses received before migration may be undocumented, unavailable to U.S. providers, or imperfectly recalled at interview, all of which would depress measured lifetime receipt even where true coverage is higher. Pneumococcal vaccination also depends on age, clinical history, prior vaccination, and provider recommendation, and the U.S. recommendations have changed repeatedly over the past two decades; differences in the likelihood of receiving a clear provider recommendation, and in the ease of establishing prior vaccination status during a routine visit, could therefore contribute. Country of origin, duration of U.S. residence, and historical rather than current access to care may also matter, since lifetime receipt accumulates over decades in which a person’s insurance and usual source of care may have differed substantially from their circumstances in 2024. The NHIS does not capture country of birth, pre-migration immunization history, or provider recommendation, so these pathways could not be examined here. What can be said is that the limited attenuation after adjustment indicates that measured differences in insurance, income, and current healthcare access do not statistically account for the disparity, which makes it a priority for dedicated study.

Foreign-born U.S. citizens fell between U.S.-born adults and noncitizens for several outcomes. This was particularly evident for pneumococcal vaccination. Citizenship may be related to greater stability in insurance coverage, healthcare access, and familiarity with the U.S. healthcare system. However, citizenship did not eliminate the pneumococcal difference. This finding also shows why combining all foreign-born adults into one group can be misleading. Foreign-born populations differ in their social circumstances and connections with the healthcare system.

The COVID-19 findings moved in the opposite direction. Foreign-born citizens and noncitizens had higher prevalence of ever receiving at least one COVID-19 vaccine dose than U.S.-born adults. This pattern remained after full adjustment. Other research provides some support for this finding. Studies conducted during the COVID-19 vaccine rollout found that foreign-born status was not necessarily associated with greater vaccine hesitancy. Frisco et al. found no significant difference in hesitancy between foreign-born Hispanic adults and U.S.-born White adults during the early vaccine rollout [42]. Montiel Ishino et al. also found that, among those who remained unvaccinated, non-U.S.-born adults reported greater willingness to seek COVID-19 vaccination than U.S.-born adults [43]. Together with our findings, these studies challenge the assumption that foreign-born populations are uniformly less receptive to vaccination.

The way COVID-19 vaccines were delivered may have contributed to this different pattern. During the pandemic, vaccination was offered in pharmacies, workplaces, public health clinics, community settings, and mass vaccination sites. Community organizations also played a substantial role. In Atlanta, a culturally and linguistically tailored program conducted 834 vaccination events in communities with large refugee and immigrant populations and administered more than 31,000 COVID-19 vaccinations [21]. In Minnesota, an established community vaccination program expanded its work during the pandemic and administered more than 43,000 COVID-19 vaccines through 1120 community-based clinics [44]. These programs show that vaccination can be brought closer to communities that may have difficulty obtaining preventive services through traditional healthcare settings. Our results may therefore reflect the broader and more diversified delivery pathways used during the COVID-19 vaccination campaign. The NHIS does not record where respondents were vaccinated or why, however, so this interpretation is a hypothesis that these data cannot test directly.

Trust, language, and community relationships also mattered during the COVID-19 response. Thomas et al. found that health departments, health systems, and community organizations serving immigrant populations emphasized trustworthy communication, equitable access, community partnerships, and culturally and linguistically appropriate approaches [20]. Salib et al. similarly found that immigrant and refugee participants experienced language barriers during COVID-19 vaccination and often relied on family members for help. Community-based organizations served as an important link to vaccination [45]. These studies do not explain the national association observed in our analysis. They do show that COVID-19 vaccination was supported by delivery approaches that differed from routine adult vaccination.

An important qualification is needed. Our COVID-19 outcome measured whether respondents had ever received at least one dose. It did not measure whether they were up to date with COVID-19 vaccination in 2024. Higher vaccination among foreign-born adults should therefore not be interpreted as evidence of higher current COVID-19 vaccine coverage. It represents cumulative receipt since COVID-19 vaccines became available. Even with this limitation, the finding is informative. Foreign-born adults were not consistently less likely to receive all vaccines. The barriers affecting routine vaccination may therefore differ from those affecting vaccination during a large public health campaign.

Duration of residence in the United States provided another useful contrast. We did not observe a clear adjusted relationship between years of U.S. residence and influenza vaccination. For COVID-19, recent immigrants had a higher adjusted prevalence of ever receiving at least one dose than immigrants who had lived in the United States for 15 years or longer. Earlier research found that recent immigrants often had lower uptake of routine adult vaccines [14,15]. The different COVID-19 pattern may reflect the unusual vaccination environment during the pandemic. Recent immigrants may have encountered vaccination through employment, community organizations, resettlement services, pharmacies, or other settings. These explanations are plausible, but they could not be tested with the NHIS data.

The interaction findings also add to the study. Insurance status modified the relationship between nativity/citizenship and influenza vaccination. Differences were relatively small among insured adults, while vaccination was lower among uninsured adults. The pattern among uninsured adults was not simply a larger disadvantage for noncitizens. Income showed a similar complexity. These findings suggest that insurance and income do not operate in exactly the same way across nativity and citizenship groups. Some foreign-born adults may obtain vaccination through community clinics, pharmacies, employers, or public health programs that are less dependent on conventional insurance. This is one possible explanation. The subgroup findings, particularly those based on smaller numbers, should be interpreted cautiously.

Our findings have implications for public health practice. Routine adult vaccination may benefit from some approaches used during the COVID-19 response. Relying mainly on preventive medical visits can miss people who do not have regular contact with the healthcare system. Pharmacies, workplaces, community health centers, mobile services, faith-based organizations, and immigrant-serving organizations can provide additional opportunities for vaccination. Community programs in Atlanta and Minnesota demonstrate how such approaches can reach populations facing traditional access barriers [21,44]. Trusted community partners can also help with language, navigation, and communication about vaccine recommendations [20,21].

The persistent pneumococcal disparity deserves particular attention. Older foreign-born adults could benefit from routine assessment of vaccination status whenever they interact with healthcare providers. Better use of immunization information systems may help when previous vaccination history is uncertain. Providers may also need support in assessing vaccination received outside the United States. Insurance coverage is important, but our findings suggest that improving insurance alone may not eliminate this disparity. Public health surveillance should also distinguish foreign-born U.S. citizens from noncitizens when sample sizes permit. Combining these populations may hide important differences.

These findings also connect directly to the migrant-health readership of this journal. Foreign-born adults in high-income destination countries are a priority population for vaccine-preventable disease control: they may have been immunized under different national schedules, may have spent part of their lives in settings where coverage of pneumococcal, influenza, or zoster vaccination is low or where these vaccines are not routinely offered to adults, and may travel to and from regions where vaccine-preventable infections remain common. Identifying which adult vaccines show persistent gaps in an immigrant population, and distinguishing gaps that track socioeconomic and healthcare access disadvantage from those that do not, is therefore relevant well beyond the United States. It informs how adult immunization programs in destination countries can be aligned with migration patterns and how catch-up vaccination might be organized for adults whose immunization histories began elsewhere.

Our study’s findings should be interpreted within the context of its several limitations. First, vaccination was self-reported and may be affected by recall. This may be particularly important for shingles and pneumococcal vaccination, which were measured as lifetime receipt. Recall may also be more difficult when vaccination occurred outside the United States. Second, the COVID-19 measure captured receipt of at least one dose ever and not current vaccination status. Third, and related, there is a temporal mismatch between the covariates and several of the outcomes. For vaccination outcomes measured as lifetime or cumulative receipt, the socioeconomic and healthcare access characteristics assessed at the 2024 interview may not reflect the conditions that prevailed when vaccination actually occurred. This mismatch is particularly relevant to the COVID-19 outcome, for which initial vaccination often preceded the survey interview by several years, and to the shingles and pneumococcal outcomes, which accumulate over decades; only the influenza outcome, which refers to the previous 12 months, is closely aligned in time with the covariates. Estimates of attenuation for the lifetime outcomes should be read with this in mind. Fourth, the NHIS does not identify immigration or visa status. The noncitizen category is therefore highly heterogeneous: it combines lawful permanent residents, temporary visa holders such as students and temporary workers, and undocumented residents, groups that differ substantially in insurance eligibility, entitlement to publicly funded programs, employment conditions, duration of residence, and exposure to immigration-related barriers to care. Estimates for noncitizens as a single category therefore average over populations whose vaccination opportunities may differ considerably, and they should not be read as describing any one of these groups. We were also unable to account for country of origin, pre-migration vaccination history, provider recommendation, vaccine confidence, health literacy, or the setting where vaccination occurred, so some residual confounding is likely to remain. In addition, the number of foreign-born noncitizens aged 65 years and older was small (160 unweighted respondents), which produced wide confidence intervals for the pneumococcal estimates in this group, and several interaction strata were similarly sparse; these estimates should be interpreted as indicating direction rather than magnitude. Finally, because the study is cross-sectional, the findings should not be interpreted as causal. Attenuation across sequential models is a description of how estimates change when covariates are added, and it can arise through several mechanisms other than mediation, including confounding, overadjustment when a covariate lies on the pathway between citizenship status and vaccination, and collider stratification when a covariate is a common effect of the exposure and the outcome [46]. Health insurance, usual source of care, and recent healthcare contact may plausibly act in more than one of these ways at once, so the attenuation reported here should not be read as a mediated proportion.

Despite these limitations, the study has several important strengths. The 2024 NHIS provides recent, nationally representative data on U.S. adults, allowing for timely assessment of adult vaccination patterns. Unlike many previous studies, we examined foreign-born U.S. citizens and foreign-born noncitizens separately rather than combining all foreign-born adults into a single category. We also evaluated four routinely recommended vaccines within the same survey and used age-appropriate analytic samples for each outcome. The analyses accounted for the complex survey design and incorporated all ten NCHS income imputations. We examined both relative and absolute differences in vaccination and then assessed how those differences changed after considering socioeconomic factors and access to healthcare. Looking across multiple vaccines also provided a broader view of vaccination patterns and highlighted disparities that may have been missed in studies focused on a single vaccine.

## 5. Conclusions

This nationally representative study showed that vaccination patterns among foreign-born adults in the United States varied substantially by vaccine and by citizenship status. Lower crude influenza and shingles vaccination among foreign-born noncitizens was markedly attenuated after adjustment for socioeconomic and healthcare access characteristics. In contrast, a substantial pneumococcal vaccination difference persisted among older foreign-born adults of both citizenship groups after full adjustment. Foreign-born adults also had a higher adjusted prevalence of ever receiving at least one COVID-19 vaccine dose, an outcome that reflects cumulative receipt since 2020 rather than current coverage.

These findings underscore the heterogeneity of adult vaccination patterns among foreign-born populations and show that lower vaccination among foreign-born adults should not be viewed as a single problem with a single explanation. Access to healthcare appears to be important for routine vaccination, but it does not statistically account for every disparity. The COVID-19 experience also shows that foreign-born populations can be reached through vaccination systems that extend beyond routine clinical care. Community-based delivery, accessible vaccination sites, trusted partnerships, and culturally and linguistically appropriate communication may have value for routine adult vaccination as well. Further investigation of structural and healthcare-related barriers is warranted, particularly for pneumococcal vaccination among older foreign-born adults, where a substantial disparity remains.

## Figures and Tables

**Table 1:** Characteristics of U.S. adults by nativity and citizenship status, National Health Interview Survey, 2024 (N = 31,362).

Characteristic	Category	Overall	U.S.-Born	Foreign-Born U.S. Citizen	Foreign-Born Noncitizen	*p* Value
Age group, years	18–29	3932 (19.9%)	3370 (21.3%)	176 (9.0%)	386 (20.5%)	<0.001
	30–49	9404 (33.4%)	7440 (31.8%)	881 (31.8%)	1083 (50.4%)	
	50–64	7459 (23.7%)	6146 (22.8%)	868 (31.8%)	445 (21.9%)	
	65–74	5784 (12.8%)	5115 (13.3%)	546 (15.6%)	123 (5.5%)	
	75+	4741 (10.2%)	4252 (10.9%)	445 (11.8%)	44 (1.6%)	
Sex	Female	16,965 (51.4%)	14,244 (51.2%)	1684 (54.5%)	1037 (48.7%)	0.003
	Male	14,392 (48.6%)	12,110 (48.8%)	1236 (45.5%)	1046 (51.3%)	
Race/ethnicity	Non-Hispanic White	21,102 (61.7%)	20,100 (72.4%)	759 (22.5%)	243 (10.4%)	<0.001
	Hispanic	4518 (17.8%)	2347 (10.9%)	975 (33.9%)	1196 (62.2%)	
	Non-Hispanic Black	3070 (11.5%)	2662 (11.9%)	288 (12.2%)	120 (7.1%)	
	Non-Hispanic Asian	1769 (6.2%)	430 (1.7%)	833 (29.2%)	506 (19.5%)	
	Non-Hispanic other/multiracial	903 (2.8%)	820 (3.1%)	65 (2.2%)	18 (0.8%)	
Education	Less than high school	2520 (10.2%)	1648 (7.4%)	320 (13.2%)	552 (32.8%)	<0.001
	High school/GED	8014 (26.8%)	6957 (27.5%)	568 (21.9%)	489 (26.4%)	
	Some college/associate	8742 (29.1%)	7813 (31.3%)	677 (25.1%)	252 (12.6%)	
	Bachelor’s or higher	11,960 (33.9%)	9860 (33.7%)	1329 (39.9%)	771 (28.2%)	
Family income-to-poverty ratio †	≥400% FPL	13,190 (42.9%)	11,336 (45.2%)	1296 (42.8%)	558 (21.8%)	—
	200–399% FPL	9371 (29.9%)	8005 (30.3%)	861 (30.6%)	505 (25.7%)	
	100–199% FPL	5549 (17.2%)	4502 (15.9%)	489 (18.0%)	558 (29.3%)	
	<100% FPL	3252 (9.9%)	2516 (8.7%)	274 (8.6%)	462 (23.2%)	
Health insurance	Insured	29,089 (90.9%)	24,867 (93.2%)	2759 (93.6%)	1463 (66.8%)	<0.001
	Uninsured	2206 (9.1%)	1439 (6.8%)	154 (6.4%)	613 (33.2%)	
Language spoken at home	English only	25,420 (76.7%)	24,287 (90.2%)	878 (27.2%)	255 (11.6%)	<0.001
	Other language at home	5923 (23.3%)	2057 (9.8%)	2040 (72.8%)	1826 (88.4%)	
U.S. Census region	Northeast	4873 (17.0%)	3992 (16.3%)	572 (22.0%)	309 (16.9%)	<0.001
	Midwest	7292 (20.8%)	6692 (22.9%)	324 (11.2%)	276 (12.3%)	
	South	11,359 (38.7%)	9517 (39.0%)	1021 (35.3%)	821 (39.9%)	
	West	7838 (23.6%)	6158 (21.7%)	1003 (31.5%)	677 (30.9%)	
Urban–rural classification	Large central metro	8945 (31.5%)	6454 (27.1%)	1420 (48.3%)	1071 (52.0%)	<0.001
	Large fringe metro	6548 (24.8%)	5385 (24.6%)	765 (29.5%)	398 (21.0%)	
	Medium/small metro	8772 (29.4%)	7751 (31.6%)	575 (18.8%)	446 (21.8%)	
	Non-metropolitan	7097 (14.3%)	6769 (16.7%)	160 (3.4%)	168 (5.3%)	
Usual place for care	Has usual place	28,713 (90.4%)	24,377 (91.6%)	2722 (92.5%)	1614 (76.6%)	<0.001
	No usual place	2616 (9.6%)	1954 (8.4%)	197 (7.5%)	465 (23.4%)	
Cost-related care barrier, past 12 months	No	28,428 (90.0%)	23,896 (90.1%)	2690 (92.0%)	1842 (86.5%)	<0.001
	Yes	2920 (10.0%)	2450 (9.9%)	229 (8.0%)	241 (13.5%)	
Saw doctor/health professional, past year	Yes	27,267 (85.2%)	23,156 (86.4%)	2587 (87.6%)	1524 (71.6%)	<0.001
	No	4043 (14.8%)	3158 (13.6%)	331 (12.4%)	554 (28.4%)	

Note. Values are unweighted n (survey-weighted %). Percentages are column percentages and exclude respondents with missing values for the characteristic shown. *p* values are from design-adjusted Rao–Scott chi-square tests of association across the three nativity/citizenship groups. FPL, federal poverty level; GED, General Educational Development credential. Urban–rural classification is the 2023 NCHS scheme. Cost-related care barrier denotes delaying or not obtaining needed medical care because of cost in the past 12 months. † Family income-to-poverty ratio is multiply imputed by NCHS. Weighted percentages are pooled across all 10 income imputations using Rubin’s rules; unweighted counts are from the first imputation because imputed category membership can differ across imputations. A single-imputation Rao–Scott *p* value is not reported for this block (—) because it would not reflect imputation uncertainty.

**Table 2:** Survey-weighted prevalence of vaccination by nativity and citizenship status, National Health Interview Survey, 2024.

Outcome	Analytic Population	Nativity/Citizenship Group	Unweighted n	Weighted % (95% CI)	*p* Value
Influenza vaccination, past 12 months	Adults ≥ 18 years	U.S.-born	26,204	43.5 (42.6–44.4)	<0.001
		Foreign-born U.S. citizen	2901	49.5 (47.2–51.8)	
		Foreign-born noncitizen	2072	32.2 (29.8–34.7)	
COVID-19 vaccination, ≥1 dose ever		U.S.-born	26,196	75.6 (74.7–76.5)	<0.001
		Foreign-born U.S. citizen	2903	88.4 (86.9–89.9)	
		Foreign-born noncitizen	2073	82.6 (80.6–84.5)	
Shingles vaccination, ever	Adults ≥ 50 years	U.S.-born	15,378	45.1 (43.9–46.2)	<0.001
		Foreign-born U.S. citizen	1821	43.2 (40.1–46.4)	
		Foreign-born noncitizen	599	24.3 (20.3–28.8)	
Pneumococcal vaccination, ever	Adults ≥ 65 years	U.S.-born	9206	67.2 (65.9–68.4)	<0.001
		Foreign-born U.S. citizen	977	55.7 (51.8–59.5)	
		Foreign-born noncitizen	160	34.5 (26.7–43.2)	

Note. CI, confidence interval. Estimates use the NHIS final annual Sample Adult weight, pseudo-stratum, and pseudo-primary sampling unit, with each outcome-specific population analyzed as a subset of the full-sample design; 95% CIs are logit-transformed. *p* values are from design-adjusted Rao–Scott chi-square tests across the three groups. Influenza and COVID-19 outcomes are assessed among adults aged ≥18 years, shingles among adults aged ≥50 years, and pneumococcal among adults aged ≥65 years; the ≥65-year pneumococcal denominator is applied to the full 2024 survey year to maintain a single consistent denominator despite the late-October 2024 change in the recommended age to ≥50 years. The COVID-19 outcome denotes ever having received at least one dose and reflects cumulative historical uptake rather than up-to-date vaccination.

**Table 3:** Fully adjusted associations between nativity/citizenship status and vaccination uptake, National Health Interview Survey, 2024.

Outcome	Nativity/Citizenship Group	Unweighted n	Adjusted Prevalence, % (95% CI)	aPR (95% CI)	aPD, Percentage Points (95% CI)	*p* Value
Influenza vaccination, past 12 months	U.S.-born	26,204	43.0 (42.2–43.9)	1.00 (reference)	Reference	
	Foreign-born U.S. citizen	2901	43.8 (41.4–46.3)	1.02 (0.96–1.08)	0.8 (−1.9 to 3.5)	0.554
	Foreign-born noncitizen	2072	46.0 (42.2–49.7)	1.07 (0.98–1.17)	2.9 (−1.1 to 7.0)	0.140
COVID-19 vaccination, ≥1 dose ever	U.S.-born	26,196	76.0 (75.2–76.8)	1.00 (reference)	Reference	
	Foreign-born U.S. citizen	2903	80.5 (78.6–82.4)	1.06 (1.03–1.09)	4.6 (2.4 to 6.7)	<0.001
	Foreign-born noncitizen	2073	90.3 (87.7–92.9)	1.19 (1.15–1.23)	14.3 (11.4 to 17.2)	<0.001
Shingles vaccination, ever	U.S.-born	15,378	44.4 (43.3–45.5)	1.00 (reference)	Reference	
	Foreign-born U.S. citizen	1821	42.7 (39.1–46.2)	0.96 (0.88–1.05)	−1.7 (−5.7 to 2.2)	0.399
	Foreign-born noncitizen	599	37.8 (31.2–44.4)	0.85 (0.71–1.02)	−6.6 (−13.5 to 0.3)	0.081
Pneumococcal vaccination, ever	U.S.-born	9206	66.6 (65.3–67.9)	1.00 (reference)	Reference	
	Foreign-born U.S. citizen	977	56.9 (52.1–61.7)	0.85 (0.78–0.94)	−9.7 (−15.1 to −4.4)	<0.001
	Foreign-born noncitizen	160	46.9 (35.7–58.1)	0.70 (0.55–0.90)	−19.7 (−31.1 to −8.3)	0.005

Note. aPD, adjusted prevalence difference versus U.S.-born adults, in percentage points; aPR, adjusted prevalence ratio; CI, confidence interval. Unweighted n is the number of respondents in each nativity/citizenship group who were eligible for the outcome and had a valid response; the fully adjusted models retained ≥99.2% of these respondents, giving model analytic samples of 30,921 (influenza), 30,918 (COVID-19), 17,661 (shingles), and 10,271 (pneumococcal) (Supplementary Table S1). Estimates are from survey-weighted modified Poisson regression models (log link, quasi-Poisson variance, design-based standard errors) adjusted for age group, sex, race/ethnicity, Census region, urbanicity, education, family income-to-poverty ratio, language spoken at home, health insurance, usual place for care, cost-related care barrier in the past 12 months, and healthcare contact in the past year. All estimates, standardized prevalences, aPDs, confidence intervals, and *p* values combine models fitted in each of the 10 NCHS family income imputations using Rubin’s rules with Barnard–Rubin degrees of freedom. Adjusted prevalences and prevalence differences are marginally standardized to the survey-weighted covariate distribution of each analytic sample, with delta-method variances pooled across imputations. *p* values refer to the aPR. Estimates for foreign-born noncitizens in the pneumococcal analysis are based on 160 unweighted respondents and are correspondingly imprecise.

## Data Availability

No new data were created in this study. The analyses were conducted using publicly available, de-identified data from the 2024 National Health Interview Survey (NHIS), provided by the National Center for Health Statistics (NCHS), Centers for Disease Control and Prevention (CDC). The 2024 NHIS public-use data files, codebooks, and supporting documentation are freely available from the NCHS website at https://www.cdc.gov/nchs/nhis/documentation/2024-nhis.html (accessed on 1 July 2026).

## Supplementary Materials

The following supporting information can be downloaded at https://www.mdpi.com/article/10.3390/tropicalmed11100282/s1: Table S1. Analytic sample sizes by outcome and model. Table S2. Definitions of NHIS variables used in the analysis. Table S3. Validation of the merge between the Sample Adult file and the 10 multiply imputed income files. Table S4. Missing data for non-income analysis variables among all Sample Adults. Table S5. Sequentially adjusted prevalence ratios for vaccination by nativity/citizenship status. Table S6a. Prespecified tests of effect modification by health insurance and family income. Table S6b. Marginally standardized vaccination prevalence by nativity/citizenship group and effect-modifier stratum. Table S7. Duration of U.S. residence and vaccination among foreign-born adults. Figure S1. Duration of U.S. residence and adjusted vaccination prevalence among foreign-born adults.

## Abbreviations

The following abbreviations are used in this manuscript:

ACIP	Advisory Committee on Immunization Practices
aPD	Adjusted prevalence difference
aPR	Adjusted prevalence ratio
CI	Confidence interval
FPL	Federal poverty level
MI	Multiple imputation
NCHS	National Center for Health Statistics
NHIS	National Health Interview Survey
PSU	Primary sampling unit
