# Supplementary material for: Vaccination Inequities Among Foreign-Born Adults in the United States: The Role of Citizenship, Socioeconomic Disadvantage, and Healthcare Access in the 2024 National Health Interview Survey
Source: Trop Med Infect Dis. 2026 Oct 5;11(10):282. doi: 10.3390/tropicalmed11100282 (PMC13709679; doi:10.3390/tropicalmed11100282)

NHIS 2024 Vaccination Inequities: Revised Supplementary Analyses

Supplementary Table S1. Analytic sample sizes

Outcome	Eligible respondents with valid outcome	Model 1 n	Model 2 n across imputations	Model 3 n across imputations
Influenza vaccination, past 12 months	31,177	31,172	31,038	30,921
COVID-19 vaccination, ≥1 dose ever	31,172	31,167	31,033	30,918
Shingles vaccination, ever	17,798	17,798	17,715	17,661
Pneumococcal vaccination, ever	10,343	10,343	10,298	10,271

Note. Unweighted respondents with a valid outcome, by nativity/citizenship group: influenza, 26,204 U.S.-born, 2,901 foreign-born U.S. citizens, 2,072 foreign-born noncitizens; COVID-19, 26,196 / 2,903 / 2,073; shingles, 15,378 / 1,821 / 599; pneumococcal, 9,206 / 977 / 160. The fully adjusted models retained ≥99.2% of these respondents in every outcome.

Supplementary Table S2. Variable definitions

Domain	NHIS_variable	Analysis_variable	Definition
Survey design	WTFA_A	weight	Final annual Sample Adult survey weight
	PSTRAT	stratum	NHIS pseudo-stratum
	PPSU	psu	NHIS pseudo-primary sampling unit
Exposure	NATUSBORN_A, CITZNSTP_A	nativity3	U.S.-born / foreign-born U.S. citizen / foreign-born noncitizen
	YRSINUS_A	duration_us	Years in U.S. among foreign-born adults
Demographic	AGEP_A	age_cat	18-29 / 30-49 / 50-64 / 65-74 / 75+
	SEX_A	sex	Female / male
	HISPALLP_A	race_ethnicity	NHIS public-use race/ethnicity recode
Socioeconomic	EDUCP_A	education	Educational attainment
	RATCAT_A	poverty_cat	Family income-to-poverty ratio, multiply imputed
	RATCAT_A	low_income	<200% versus ≥200% FPL, multiply imputed
Marginalization	NOTCOV_A	insurance	NCHS-edited uninsured/insured status
	LANGHM_A	language_home	Language other than English spoken at home
Geography	REGION	region	Northeast/Midwest/South/West
	URBRRL23	urbanicity	2023 NCHS urban-rural classification
Access	USUALPL_A	usual_care	Has a usual place for care
	MEDDL12M_A / MEDNG12M_A	cost_barrier	Delayed or forgone care because of cost
	LASTDR_A	doctor_past_year	Saw doctor/health professional in past year
Outcome	SHTFLU12M_A	flu_vax	Influenza vaccination in past 12 months
	SHTCVD191_A	covid_vax	At least one COVID-19 vaccine dose ever
	SHTSHINGL1_A	shingles_vax	Ever received shingles vaccination
	SHTPNUEV_A	pneumo_vax	Ever received pneumococcal vaccination

Note. Variable names are reproduced exactly as published in the 2024 NHIS Sample Adult public-use file and were re-verified against the 2024 public-use codebook.

Supplementary Table S3. Multiple-imputation merge validation

Imputation	Income records	Merged adult records	Missing RATCAT_A after merge	Missing poverty category
1	32,629	32,629	0	0
2	32,629	32,629	0	0
3	32,629	32,629	0	0
4	32,629	32,629	0	0
5	32,629	32,629	0	0
6	32,629	32,629	0	0
7	32,629	32,629	0	0
8	32,629	32,629	0	0
9	32,629	32,629	0	0
10	32,629	32,629	0	0

Supplementary Table S4. Missingness of non-income analysis variables

Analysis variable	Missing, n	Missing, %
duration_us	27,690	84.9
shingles_vax	13,598	41.7
pneumo_vax	1,284	3.9
nativity3	1,267	3.9
language_home	1,228	3.8
covid_vax	514	1.6
flu_vax	497	1.5
cost_barrier	278	0.9
doctor_past_year	256	0.8
usual_care	239	0.7
education	147	0.5
insurance	93	0.3
age_cat	52	0.2
sex	5	0.0
race_ethnicity	0	0.0
region	0	0.0
urbanicity	0	0.0

Supplementary Table S5. Sequentially adjusted prevalence-ratio models

Outcome	Model	Nativity/citizenship group	Analytic n	aPR (95% CI)	P value
Influenza vaccination, past 12 months	Model 1	U.S.-born	31,172	1.00 (reference)	
	Model 1	Foreign-born U.S. citizen	31,172	0.98 (0.92-1.03)	0.410
	Model 1	Foreign-born noncitizen	31,172	0.84 (0.77-0.92)	<0.001
	Model 2	U.S.-born	31,038	1.00 (reference)	
	Model 2	Foreign-born U.S. citizen	31,038	1.01 (0.95-1.08)	0.654
	Model 2	Foreign-born noncitizen	31,038	0.92 (0.84-1.00)	0.060
	Model 3	U.S.-born	30,921	1.00 (reference)	
	Model 3	Foreign-born U.S. citizen	30,921	1.02 (0.96-1.08)	0.554
	Model 3	Foreign-born noncitizen	30,921	1.07 (0.98-1.17)	0.140
COVID-19 vaccination, ≥1 dose ever	Model 1	U.S.-born	31,167	1.00 (reference)	
	Model 1	Foreign-born U.S. citizen	31,167	1.06 (1.03-1.08)	<0.001
	Model 1	Foreign-born noncitizen	31,167	1.08 (1.04-1.11)	<0.001
	Model 2	U.S.-born	31,033	1.00 (reference)	
	Model 2	Foreign-born U.S. citizen	31,033	1.06 (1.03-1.08)	<0.001
	Model 2	Foreign-born noncitizen	31,033	1.13 (1.10-1.17)	<0.001
	Model 3	U.S.-born	30,918	1.00 (reference)	
	Model 3	Foreign-born U.S. citizen	30,918	1.06 (1.03-1.09)	<0.001
	Model 3	Foreign-born noncitizen	30,918	1.19 (1.15-1.23)	<0.001
	Model 1	U.S.-born	17,798	1.00 (reference)	

Outcome	Model	Nativity/citizenship group	Analytic n	aPR (95% CI)	P value
Shingles vaccination, ever	Model 1	Foreign-born U.S. citizen	17,798	0.92 (0.85-1.01)	0.084
	Model 1	Foreign-born noncitizen	17,798	0.64 (0.54-0.77)	<0.001
	Model 2	U.S.-born	17,715	1.00 (reference)	
	Model 2	Foreign-born U.S. citizen	17,715	0.96 (0.88-1.06)	0.444
	Model 2	Foreign-born noncitizen	17,715	0.76 (0.63-0.91)	0.004
	Model 3	U.S.-born	17,661	1.00 (reference)	
	Model 3	Foreign-born U.S. citizen	17,661	0.96 (0.88-1.05)	0.399
	Model 3	Foreign-born noncitizen	17,661	0.85 (0.71-1.02)	0.081
Pneumococcal vaccination, ever	Model 1	U.S.-born	10,343	1.00 (reference)	
	Model 1	Foreign-born U.S. citizen	10,343	0.83 (0.76-0.91)	<0.001
	Model 1	Foreign-born noncitizen	10,343	0.57 (0.45-0.73)	<0.001
	Model 2	U.S.-born	10,298	1.00 (reference)	
	Model 2	Foreign-born U.S. citizen	10,298	0.86 (0.78-0.94)	0.001
	Model 2	Foreign-born noncitizen	10,298	0.63 (0.49-0.81)	<0.001
	Model 3	U.S.-born	10,271	1.00 (reference)	
	Model 3	Foreign-born U.S. citizen	10,271	0.85 (0.78-0.94)	<0.001
	Model 3	Foreign-born noncitizen	10,271	0.70 (0.55-0.90)	0.005

Model 1 is fitted once because it contains no imputed income variable.
Models 2 and 3 are fitted in all 10 NCHS income imputations and pooled using Rubin's rules.

Supplementary Table S6a. Prespecified interaction tests

Outcome	Modifier	Analytic n	Global interaction P
Influenza vaccination, past 12 months	Health insurance	30,921	<0.001
Influenza vaccination, past 12 months	Family income (<200% vs ≥200% FPL)	30,921	<0.001
Shingles vaccination, ever	Health insurance	17,661	0.022
Shingles vaccination, ever	Family income (<200% vs ≥200% FPL)	17,661	0.168

Interaction tests are joint Wald tests of Rubin-pooled interaction coefficients and covariance matrices.
Both insurance and family-income interaction analyses pool all 10 income imputations.

Supplementary Table S6b. Marginal standardized prevalence by interaction stratum

Outcome	Modifier	Nativity/citizenship group	Modifier level	Adjusted prevalence, % (95% CI)	Interaction P	Analytic n
Influenza vaccination, past 12 months	Health insurance	U.S.-born	Insured	44.2 (43.3-45.1)	<0.001	30,921
			Uninsured	19.3 (16.0-22.6)	<0.001	30,921
		Foreign-born U.S. citizen	Insured	44.5 (42.0-46.9)	<0.001	30,921
			Uninsured	32.3 (19.7-44.8)	<0.001	30,921
		Foreign-born noncitizen	Insured	45.0 (41.1-48.9)	<0.001	30,921
			Uninsured	32.0 (25.3-38.7)	<0.001	30,921
	Family income	U.S.-born	≥200% FPL	44.3 (43.3-45.3)	<0.001	30,921
			<200% FPL	38.7 (37.1-40.4)	<0.001	30,921
		Foreign-born U.S. citizen	≥200% FPL	42.9 (40.3-45.6)	<0.001	30,921
			<200% FPL	45.5 (41.1-50.0)	<0.001	30,921
		Foreign-born noncitizen	≥200% FPL	41.6 (37.3-45.9)	<0.001	30,921
			<200% FPL	49.6 (43.6-55.6)	<0.001	30,921
Shingles vaccination, ever	Health insurance	U.S.-born	Insured	45.0 (43.8-46.1)	0.022	17,661
			Uninsured	15.0 (9.1-20.9)	0.022	17,661
		Foreign-born U.S. citizen	Insured	42.9 (39.2-46.5)	0.022	17,661
			Uninsured	33.3 (9.7-56.9)	0.022	17,661

Outcome	Modifier	Nativity/citizenship group	Modifier level	Adjusted prevalence, % (95% CI)	Interaction P	Analytic n
		Foreign-born noncitizen	Insured	36.5 (29.8-43.2)	0.022	17,661
			Uninsured	26.5 (12.5-40.6)	0.022	17,661
	Family income	U.S.-born	≥200% FPL	46.7 (45.4-48.0)	0.168	17,661
			<200% FPL	36.1 (33.9-38.2)	0.168	17,661
		Foreign-born U.S. citizen	≥200% FPL	44.1 (40.1-48.1)	0.168	17,661
			<200% FPL	37.3 (31.8-42.8)	0.168	17,661
		Foreign-born noncitizen	≥200% FPL	35.1 (26.8-43.4)	0.168	17,661
			<200% FPL	36.4 (27.1-45.8)	0.168	17,661

Supplementary Table S7. Duration of U.S. residence among foreign-born adults

Outcome	Years lived in U.S.	Analytic n	Adjusted prevalence, % (95% CI)	aPR (95% CI)	P value
Influenza vaccination, past 12 months	≥15 years	4,846	42.7 (40.9-44.6)	1.00 (reference)	
	5-14 years	4,846	39.2 (34.6-43.9)	0.92 (0.80-1.05)	0.197
	<5 years	4,846	38.3 (31.5-45.2)	0.90 (0.74-1.08)	0.256
COVID-19 vaccination, ≥1 dose ever	≥15 years	4,851	84.9 (83.4-86.5)	1.00 (reference)	
	5-14 years	4,851	87.4 (84.6-90.2)	1.03 (0.99-1.07)	0.155
	<5 years	4,851	93.9 (90.0-97.8)	1.11 (1.05-1.16)	<0.001

All adjusted duration estimates pool all 10 income imputations.

Supplementary Figure S1. Duration of U.S. residence and vaccination uptake

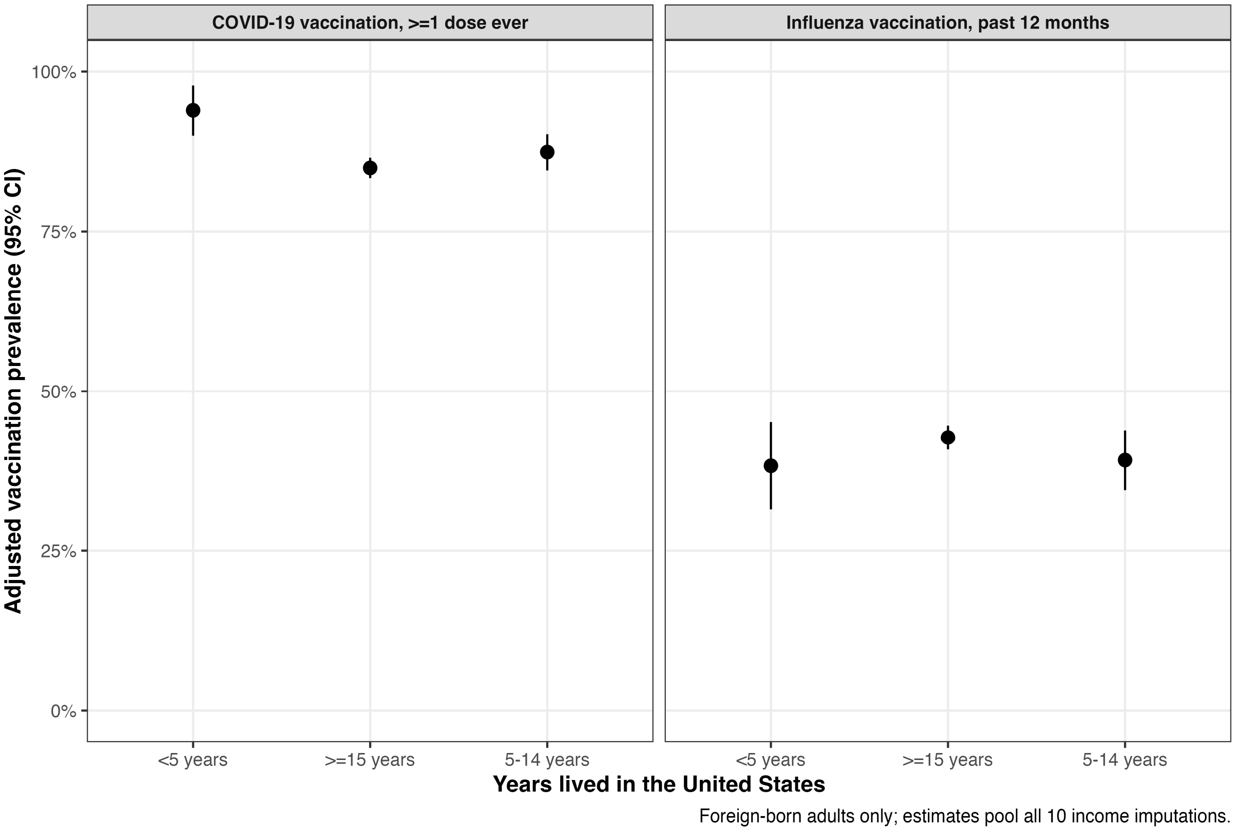

Supplement: Supplementary file 1 [file tropicalmed-11-00282-s001.zip › tropicalmed-4589170-supplementary.pdf]
